# The FAMULATUR PLUS as an innovative approach for teaching physical examination skills

**DOI:** 10.3205/zma001003

**Published:** 2016-02-15

**Authors:** Achim Jerg, Wolfgang Öchsner, Henriette Wander, Harald C. Traue, Lucia Jerg-Bretzke

**Affiliations:** 1 University Hospital Ulm, Department of Psychosomatic Medicine and Psychotherapy, Medical Psychology, Ulm, Germany; 2 University Hospital Ulm, Department for Cardiac Anesthesiology, Ulm, Germany; 3 University of Ulm, Office of the Dean of Medical Studies, Ulm, Germany

**Keywords:** clinical skills, medical education, physical examination, practical training

## Abstract

The FAMULATUR PLUS is an innovative approach to teaching physical examination skills. The concept is aimed at medical students during the clinical part of their studies and includes a clinical traineeship (English for “Famulatur”) extended to include various courses (“PLUS”). The courses are divided into clinical examination courses and problembased-learning (PBL) seminars. The concept’s special feature is the full integration of these courses into a 30-day hospital traineeship. The aim is to facilitate the transfer of knowledge from the courses into daily practice. Each week of the FAMULATUR PLUS is structured in line with the courses and focuses on a particular part of the body (e.g., abdomen). A physical examination course under the supervision of a physician is offered at the beginning of the week. Here, medical students learn the relevant examination techniques by practicing on each other (partner exercises). Subsequently, the techniques taught are applied independently during everyday work on the ward, corrected by the supervisor, if necessary, and thereby reinforced. The final POL seminar takes place towards the end of the week. Possible differential diagnoses are developed based on a clinical case study. The goal is to check these by taking a fictitious medical history and performing a physical examination, as well as to make a preliminary diagnosis. Finally, during the PBL seminar, medical students will be shown how physical examination techniques can be efficiently applied in the diagnosis of common cardinal symptoms (e.g., abdominal pain). The initial implementation of the FAMULATUR PLUS proved the practical feasibility of the concept. In addition, the accompanying evaluation showed that the participants of the pilot project improved with regard to their practical physical examination skills.

## Zusammenfassung

Die FAMULATUR PLUS ist ein innovativer Ansatz zur Vermittlung praktischer Fertigkeiten in der körperlichen Untersuchung. Das Konzept richtet sich an Medizinstudierende im klinischen Studienabschnitt und beinhaltet eine um Lehrveranstaltungen („PLUS“) erweiterte Famulatur. Die Lehrveranstaltungen umfassen klinische Untersuchungskurse und problemorientiertes Lernen (POL). Besonderheit des Konzepts ist die vollständige Integration dieser Lehrveranstaltungen in eine 30tägige Krankenhausfamulatur. Dadurch soll der Wissenstransfer von den Lehrveranstaltungen in die tägliche Praxis vereinfacht werden. Jede einzelne Woche der FAMULATUR PLUS ist hinsichtlich der Lehrveranstaltungen strukturiert vorgeplant und steht im Zeichen eines Körperabschnitts (z.B. Abdomen). Zu Wochenbeginn wird ein Untersuchungskurs unter ärztlicher Leitung angeboten. Hier erlernen die Medizinstudierenden die relevanten Untersuchungstechniken zunächst durch gegenseitige Anwendung (Partnerübungen). Anschließend werden die gelehrten Techniken im Stationsalltag eigenverantwortlich angewandt, gegebenenfalls durch den Betreuer nochmals korrigiert, und somit gefestigt. Gegen Ende der Woche findet dann das abschließende POL-Seminar statt. Ausgehend von einem klinischen Fallbeispiel werden hier mögliche Differentialdiagnosen erarbeitet. Diese gilt es dann durch fiktive Anamneseerhebung und vor allem körperliche Untersuchung zu überprüfen sowie eine Verdachtsdiagnose zu stellen. Schlussendlich soll den Medizinstudierenden im POL gezeigt werden, wie Techniken der körperlichen Untersuchung bei der Diagnostik häufiger Leitsymptome (z.B. Bauchschmerzen) effizient angewandt werden können. Bei der ersten Durchführung der FAMULATUR PLUS als Pilotprojekt erwies sich die praktische Umsetzbarkeit des Konzepts. Zudem zeigte die begleitende Evaluation eine Verbesserung der Teilnehmer des Pilotprojekts hinsichtlich der praktischen Fertigkeiten in der körperlichen Untersuchung.

## 1. Introduction

The physical examination of patients – alongside the assessment of their medical history – is an essential medical skill. Approximately three quarters of all diagnoses can be made based solely on the medical history and physical examination [1], [2]. Despite this finding, the physical examination is increasingly supplanted by the advent of instrument-based diagnostics and may therefore even appear “outdated”. It is thus hardly surprising that teaching medical students physical examination skills now frequently takes a back seat. This observation is supported primarily by publications from the USA. Unanimously, these publications lead to disillusionment with regard to the physical examination skills of medical students and physicians. For example, scientific studies have shown that medical students, as well as residents and specialists, were often unable to correctly evaluate common auscultation findings of the lungs and heart [3], [4], [5]. Medical students were hardly able to carry out a complete general internist examination [6]. Although the previously introduced works originate abroad, similar knowledge deficits must be assumed to exist among German medical students [7]. 

## 2. Objective

The objective of the project presented here is the intensive transfer of physical examination skills to medical students. This should be done as part of a clinical traineeship, which is complemented by an integrated teaching program (FAMULATUR PLUS). 

## 3. Pilot project and evaluation

From the perspective of a medical student, a clinical traineeship is the practical addition to his/her theoretical studies. The FAMULATUR PLUS was developed based on this conception and is composed of three didactic elements – the physical examination course, the clinical traineeship, and the PBL seminar – described in more detail below. The temporal sequence of the didactic elements follows a standardized structure (see 3.4). 

### 3.1. Physical Examination Course

**Figure 1:**
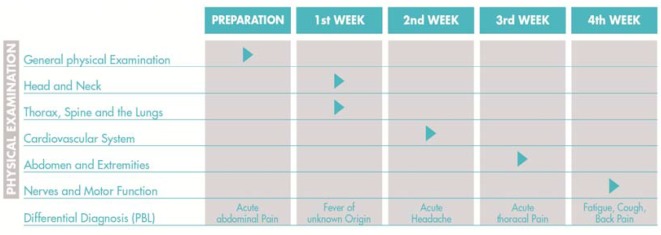
A course on general internal examination (“General physical Examination”) and a problembased-learning (PBL) seminar on the subject of “Acute abdominal Pain” take place prior to the start of the actual clinical traineeship. The subsequent examination courses build on the “General physical Examination” course and look at specific organs and structures in more detail. The PBL seminars focus on initial and differential diagnoses based on clinical case studies.

The FAMULATUR PLUS includes a total of five physical examination courses. These courses are intended to take approximately 120 minutes each and are taught by physicians. The teaching and learning objectives of all physical examination courses are guided by the current literature [7], [8], [9], [10], [11], [12], [13]. The first physical examination course takes place prior to the clinical traineeship and focuses on the general internist examination (“General physical Examination”). It builds on and refreshes the knowledge that medical students have acquired in the physical examination courses offered at their respective universities. In addition, it aims to teach the medical students how to examine the whole body of a patient in a time-efficient manner. The subsequent physical examination courses “Head and Neck, Thorax, Spine and the Lungs”, “Cardiovascular System”, “Abdomen and Extremities”, as well as “Nerves and Motor Function” thematically build on the general internist examination course and extend the examination techniques to particular organs and structures (see Figure 1 (Fig. 1)). The “General physical Examination” course includes an initial examination of the abdomen, for example, but more detailed examination techniques (e.g., Carnett’s test) are introduced only in the “Abdomen and Extremities” course. 

Although the emphases of the physical examination courses vary depending on the instructor and the topic in question, this does not apply to the curricular structure. The sequence of the physical examination courses is thus always the same and can be divided into four steps – demonstration, deconstruction, comprehension, and implementation – following a modified Peyton’s technique [14], [15]. By way of introduction, the examination techniques to be learned are demonstrated in their usual sequence and at the regular pace by the physician in charge of teaching the course (demonstration). Subsequently, the physician divides the respective examination technique into individual actions (deconstruction). The aim of deconstruction is to render the examination technique more easily reproducible for the medical students. To check whether the examination technique taught was actually understood, the physician asks the medical students to describe the individual steps while he/she carries them out. Thus, in a sense, the physician is now being instructed by the medical students. This promotes an accurate mental representation of the procedure and enables faster learning and improved recall (comprehension). During the last part of the physical examination course, the medical students are responsible for learning the examination techniques through mutual application (implementation). The physician teaching the course is available to answer questions. The physician is further responsible for taking corrective action with regard to the practical application of the examination techniques, if necessary.

#### 3.2. Clinical traineeship

The clinical traineeship is a key component of the proposed concept, seeing as it allows the transfer of knowledge from the physical examination courses to the daily work on the ward. Every week, practical exercises are assigned to encourage the medical students to apply the learned examination techniques. These exercises can be completed flexibly by the medical students. Every week, at least three general internist and three organ-specific examinations, which correspond to the respective physical examination course, are to be completed. If the examination course “Head and Neck, Thorax, Spine and the Lungs” took place in the first week, for example, the medical students are required to complete three general internal examinations and three examinations of head, neck, thorax, spine, and lungs in the corresponding week. This procedure also applies to all other weeks of the clinical traineeship. The medical students receive medical history and examination forms on which they can briefly document their examination findings. The completed medical history and examination forms are submitted to the ward physician for review and must be countersigned by him/her. The review of the medical history and examination forms ensures that

1. the medical students are “forced” to physically examine the patients and
2. difficulties in the practical implementation of the examination techniques are revealed and can serve as the basis for the optional feedback session with the ward physician (see 3.4).

In addition, the ward physician is called upon to provide the medical students with constructive feedback regarding their examination skills as well as to identify opportunities for improvement on a weekly basis. Furthermore, the medical students are free to check their own performance by comparing their examination findings with the medical findings documented in the patient’s chart. 

#### 3.3. Problembased-learning

Problembased-learning (PBL) constitutes the third didactic element alongside the physical examination courses and clinical traineeship. Five PBL seminars take place during the FAMULATUR PLUS. Numerous differential diagnoses are initially postulated based on a clinical case study. The number of differential diagnoses is to be gradually reduced down to a concrete diagnosis on the basis of a fictitious anamnesis and physical examination. After completion of the PBL, the medical students are responsible for independently performing follow-up course work. Such follow-up work may, for example, consist of developing a diagnostic algorithm for a particular cardinal symptom (e.g., acute abdominal pain). On the one hand, the PBL and follow-up serve to show the importance of the physical examination for the diagnostic process. On the other hand, the PBL teaching method is well suited to improve the clinical thinking of the medical students by practicing problem solving processes [15], [16]. As was the case with the first physical examination course (“General physical Examination”), the first PBL seminar also takes place before the actual clinical traineeship starts. Apart from dates and topics, PBL seminars do not differ in terms of their curricular structure. The individual PBL cases were compiled based on the seven steps of PBL [17], [18]. A detailed overview of the course contents can be found in Figure 1 (Fig. 1).

Student tutors are responsible for teaching and moderating the PBL seminar. Starting from the summer semester of 2015, didactically trained tutors (“peer teachers”) from the “Train the Tutor” training program of the Faculty of Medicine at the University of Ulm will be used in the FAMULATUR PLUS [http://fakultaet.medizin.uni-ulm.de/fileadmin/Studiengaenge/Humanmedizin/Informationen_Lehren-Lernen-Track_SS15.pdf; accessed on 2 December 2014].

#### 3.4. Integration and sequence of courses

**Figure 2:**
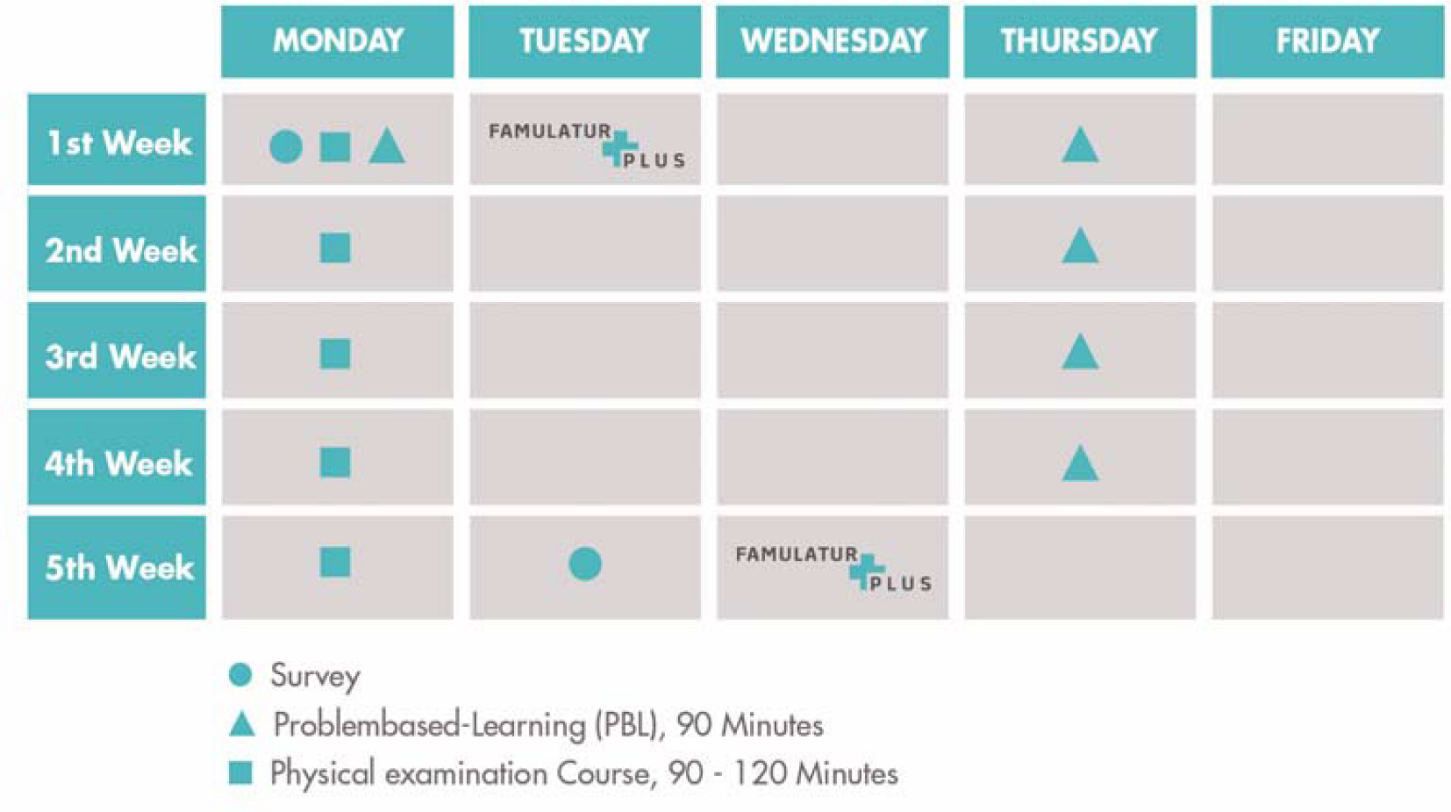
The FAMULATUR PLUS starts with a preparation day during which the first examination course and the first problembased-learning (PBL) seminar take place. Thereafter, the PBL seminars take place every Thursday and the examination courses every Monday.

The didactic elements follow a standard chronological pattern (see Figure 2 (Fig. 2)). As a rule, all courses are integrated into the regular clinical traineeship, with the exception of the “General physical examination” course and the “Acute abdominal Pain” PBL seminar. These two courses take place in advance during an introduction day. The introduction day is necessary, due to the accompanying scientific survey of the FAMULATUR PLUS (see 4.). This study aims to evaluate whether there is an improvement in the practical examination skills of the medical students based on findings from two time points before (introduction day) and after the FAMULATUR PLUS.

The examination courses are held on the same weekday every week and, with the exception of the “General physical Examination” course, deal with specific organs and structures (see 3.1). The organ- or structure-specific examination courses build on the “General physical Examination” course, going more in-depth into selective topics. During the following week, the medical students have the opportunity to apply the examination techniques learned in practice and work on their assignments. These assignments consist of three general internist examinations and three organ- and structure-specific examinations. The latter always correspond to the examination course of the current clinical traineeship week (see 3.3). The PBL seminar also takes place on a weekly basis. Here, medical students can practice ascertaining tentative and differential diagnoses by using primarily fictitious medical histories and physical examinations based on real clinical cases. Each clinical traineeship week concludes with a short meeting with the ward physician. During this meeting, the medical students should be given constructive feedback on their examination skills based on their reviewed medical history and examination forms. The meeting is optional and should be actively sought by the medical students. The retrospective, as opposed to immediate, feedback meeting aims to avoid making the medical students feel “monitored,” which may have otherwise impaired their individual learning development [17].

## 4. Pilot project and evaluation

The FAMULATUR PLUS was first implemented as a pilot project comprising five medical students in August 2014. For this purpose, a collaboration with the Department of Internal Medicine at Donauklinik Neu-Ulm took place. The pilot project was intended to answer two key questions:

1. Can the concept be implemented in practice?
2. Have the medical students’ practical physical examination skills improved after the FAMULATUR PLUS?

The FAMULATUR PLUS pilot project was a success with regard to its practicability, both among students and instructors. There were no time conflicts between courses and responsibilities in terms of patient care on the ward. Furthermore, students perceived the courses as a welcome change from their everyday work on the ward. The incorporation of instructors was achieved without problems. In addition, the instructors adhered strictly to the catalog of learning objectives and taught – as desired – through practical demonstrations and exercises. Neither medical students nor instructors requested changes with regard to content or organizational structure. Thus, no changes had to be made. Furthermore, since the feasibility of the concept was confirmed, the acquisition of additional cooperating hospitals was set in motion. 

A scientific study was initiated to answer the second question. This study aims to examine whether the medical students’ practical physical examination skills have improved after completion of the FAMULATUR PLUS. This is achieved by means of a self-assessment questionnaire and the heart and lung auscultation tests (auscultation tests) described in the literature [4], [5], as well as through a physical examination (physical examination test) [6]. Both the questionnaire and the auscultation and physical examination tests are assessed before and after the FAMULATUR PLUS. The questionnaire measures the medical students’ individual and subjective assessment of their practical physical examination skills. By contrast, the auscultation and physical examination tests objectively verify the practical physical examination skills of the medical students. During the physical examination test the medical students are asked to perform a general internist examination on a simulation patient. The physical examination test is recorded anonymously by video camera and subsequently evaluated by a neutral physician according to a standardized catalog. The physical examination test is evaluated based on a complete and correct implementation of the examination techniques required by the literature [19]. The auscultation test focuses on the medical students’ skills with regard to the acoustic evaluation of heart and lung sounds. The auscultation tests are also conducted anonymously. Again, a neutral physician reviews the correct diagnosis of the auscultation examples. As mentioned elsewhere, the auscultation and physical examination tests are carried out using established methods [4], [5], [6].

The evaluation of the pilot sample, comprising five medical students, showed some initial trends: for example, the medical students’ average assessment of their practical physical examination skills in the advance questionnaire corresponded to a grade of 4 (with 1 being the best and 6 being the worst), while their average assessment after completion of the FAMULATUR PLUS corresponded to a grade of 2. A similar picture emerged for the auscultation and physical examination tests. Across tests, approximately 75 percent of the requirements were met by the medical students after completion of the FAMULATUR PLUS; prior to completion, only approx. 26 percent were met.

## 5. Conclusion and outlook

In an initial pilot project, the FAMULATUR PLUS proved to be a feasible teaching method for imparting physical examination skills. Transfer of the concept to other hospitals is planned. Furthermore, potential benefits of the FAMULATUR PLUS for the practical training of medical students are being discussed. Evaluation of previous data showed an improvement in the practical physical examination skills of participating medical students. However, the significance of these results is limited given the small sample size and lack of a control group. As a result of this, modification of the study design as a randomized and controlled study appears sensible [20]. Moreover, the question arises to what extent the transfer of knowledge from the examination courses into clinical practice is actually successful. Published methodological approaches considering similar issues exist that could be used to clarify this point in the further course of the project [21], [22], [23].

## Competing interests

The authors declare that they have no competing interests.
